# Topical Mucoadhesive Alginate-Based Hydrogel Loading Ketorolac for Pain Management after Pharmacotherapy, Ablation, or Surgical Removal in Condyloma Acuminata

**DOI:** 10.3390/gels7010008

**Published:** 2021-01-23

**Authors:** Salima El Moussaoui, Francisco Fernández-Campos, Cristina Alonso, David Limón, Lyda Halbaut, Maria Luisa Garduño-Ramirez, Ana Cristina Calpena, Mireia Mallandrich

**Affiliations:** 1 Departament de Farmàcia, Tecnologia Farmacèutica i Fisicoquímica, Faculty of Pharmacy and Food Sciences, University of Barcelona, Av. Joan XXIII 27-31, 08028 Barcelona, Spain; selmouel9@alumnes.ub.edu (S.E.M.); halbaut@ub.edu (L.H.); anacalpena@ub.edu (A.C.C.); 2 Reig-Jofre Laboratories, Av. de les Flors s/n, 08970 Sant Joan Despí, Spain; ffernandez@reigjofre.com; 3 Institute of Advanced Chemistry of Catalonia-CSIC (IQAC-CSIC), 18-26 Jordi Girona St, 08034 Barcelona, Spain; cristina.alonso@iqac.csic.es; 4 Departament de Farmacologia, Toxicologia i Química Terapèutica, Faculty of Pharmacy and Food Sciences, Universitat de Barcelona, Av. Joan XXIII 27-31, 08028 Barcelona, Spain; davidlimon@ub.edu; 5 Institut de Nanociència i Nanotecnologia IN2UB, Universitat de Barcelona, 08028 Barcelona, Spain; 6 Centro de Investigaciones Químicas, Universidad Autónoma del Estado, de Morelos, Avenida Universidad 1001, Cuernavaca 62209, Morelos, Mexico; lgarduno@uaem.mx

**Keywords:** *Condyloma acuminata*, ketorolac, pain management, alginate hydrogel, topical delivery, transdermal, mucosal delivery, mucoadhesive, anti-inflammatory

## Abstract

Condyloma acuminata is an infectious disease caused by the human papilloma virus (HPV) and one of the most common sexually transmitted infections. It is manifested as warts that frequently cause pain, pruritus, burning, and occasional bleeding. Treatment (physical, chemical, or surgical) can result in erosion, scars, or ulcers, implying inflammatory processes causing pain. In this work, a biocompatible topical hydrogel containing 2% ketorolac tromethamine was developed to manage the painful inflammatory processes occurring upon the removal of anogenital condylomas. The hydrogel was physically, mechanically, and morphologically characterized: it showed adequate characteristics for a topical formulation. Up to 73% of ketorolac in the gel can be released following a one-phase exponential model. Upon application on human skin and vaginal mucosa, ketorolac can permeate through both of these and it can be retained within both tissues, particularly on vaginal mucosa. Another advantage is that no systemic side effects should be expected after application of the gel. The hydrogel showed itself to be well tolerated in vivo when applied on humans, and it did not cause any visible irritation. Finally, ketorolac hydrogel showed 53% anti-inflammatory activity, suggesting that it is a stable and suitable formulation for the treatment of inflammatory processes, such as those occurring upon chemical or surgical removal of anogenital warts.

## 1. Introduction

Condyloma acuminata (CA) is one of the most common sexually transmitted infections [1] caused by various genotypes of Human Papilloma Virus (HPV) [2]. There are more than 180 genotypes described, around 40 of which have been related to anogenital warts, most particularly genotypes 6 and 11 [3]. Genotypes 16 and 18 attracted attention because of their oncogenic nature and they have been associated to vaginal cervix cancer and anus cancer [4].

Although the introduction of the tetravalent vaccine against HPV caused a significant reduction in its incidence [5], the disease incidence is still high, especially in certain countries with socio-economic issues [5]. CA can affect both sexes at any age, being the maximum incidence is between 20- and 24-year-old women and between 25- and 29-year-old men [6]. Symptoms associated to CA depend on anatomical location, number, and size of the lesions [3], being pruritus the most frequent, followed by leukorrhea, discomfort, bleeding and pain. In addition, CA usually involves psychological aspects such as concern, shame, and lack of self-esteem in the sexual sphere [1].

On some occasions CA can be resolved spontaneously or will remain with no change, but in other cases lesions need to be treated, specially to prevent future outcomes such as cancer. In these cases, a therapeutic intervention should be carried out, considering location of the disease, number of warts, and patient related aspects such as life-style, and economic considerations, among others [6]. Several theories are described in the literature but there is not enough scientifical evidence demonstrating one treatment to be superior over others [7]. Pharmacological treatment involves an immunomodulatory approach (topical imiquimod, podophyllotoxin, and sinecatechins of tea extract), physical agents (cryotherapy, trichloroacetic acid, electrocoagulation, and CO_2_ laser), and/or surgery. Other treatments backed up less scientific evidence due to the few clinical trials, involve intralesional interferon, photodynamic therapy, and topical cidofovir [8]. Unfortunately, when there is intravaginal or intra-anal CA, the treatment alternatives are reduced, and on some occasions, surgery is the only option.

Pain management is under extensive study so that it may improve patient quality of life and reduce the post-surgery discomfort. Ambulatory and minor surgeries usually combine anesthetics, analgesics, and anti-inflammatory drugs in different pre, intra, or post-operative regimes. Ketorolac is a non-steroidal anti-inflammatory drug with potent analgesic properties close to those of opioids, with no addictiveness potential and no sedative properties [9]. Several authors injected surgery ketorolac at the anatomical site (colon or vaginal), with local effect, in haemorrhoidectomy [10] and other colon and vaginal surgical procedures [11]. It resulted in successful pain management and improvement in the patient’s recovery. Ketorolac demonstrated to be effective in the management of postoperative pain when administrated in a single dose [12]. It is also administrated topically as preoperative medication in eye laser keratectomy and cataracts [13,14,15]. In fact, one of the most common applications of topical ketorolac has been in ophthalmology, with very low topical administration in skin and mucosa. Considering the successful results in pain management after local injection in colon and vaginal surgeries, as previously described, topical ketorolac is proposed is proposed for intravaginal application, pre-vaginal applications and/or anal application in CA removal.

Two main events modulate the successful topical delivery: diffusion through the vehicle towards the surface of the tissue (in this case, skin or vaginal mucosa) and the partitioning between the vehicle and the tissue. For this reason, several aspects should be considered during the development. On one hand, the physicochemical properties of the drug modulate its intrinsic permeability, being the molecular weight and the log P (octanol–water partitioning) the main parameters used to predict successful permeation. On the other hand, characteristics of the formulation also modulate the permeation, such as the complete solution of the drug in the vehicle, the particle size (in suspensions), pH, relative polarity between formulation-drug-body surface, and the presence of permeation enhancers.

As well, the biocompatibility of the formulation is essential for treatment tolerance and patient compliance. Hydrogels are traditional formulations composed of different polymers types, natural ones (different cellulose grades and other polysaccharides) or synthetic ones (polyacrylates, polyvinyl pyrrolidone, etc.) All these polymers are able to retain water in their 3D network by creating hydrogen bonds between water molecules and polymer molecules. Alginate is a natural linear copolymer formed by (1,4)-β-D-mannuronic acid and α-L-guluronic acid residues, which can be arranged as homopolymeric sequences of each residue, or with alternating residues. Alginate-based gels show interesting mucoadhesive properties and a very good compatibility profile [16], having been extensively used in topical drug delivery systems with successful results.

**Figure 1:**
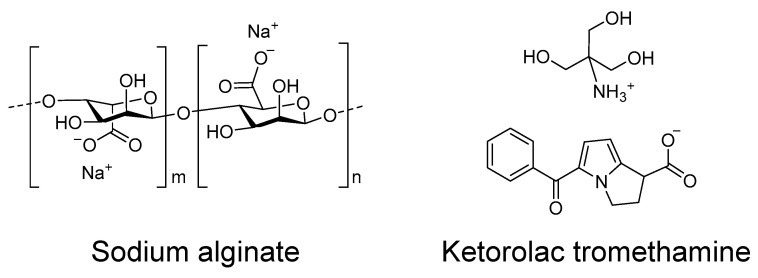
Chemical structures of sodium alginate polymer and ketorolac tromethamine.

In this work, we designed and developed a hydrogel using sodium alginate as the gelator polymer and ketorolac tromethamine (Figure 1) as a potent non-steroidal anti-inflammatory drug, as a candidate for the local pre- or post-treatment of inflammatory processes derived from CA surgical removal. The hydrogel has been extensively characterized in terms of appearance, microscopic morphology, and mechanical properties, among others. As well as this, its biopharmaceutical properties have also been investigated in terms of drug release, drug permeation across the skin and vaginal mucosa, and the amount of drug retained within the tissue after application for performing a local therapeutical activity. Moreover, the biocompatibility of the formulation has been tested by measuring the trans-epidermal water loss (TEWL) in humans, and the anti-inflammatory efficacy of the ketorolac hydrogel (KT hydrogel) has been evaluated in mice. Results from this work show that this hydrogel is a promising candidate for the described indication.

## 2. Results and Discussion

### 2.1. Physical Characterization of KT Hydrogel

#### 2.1.1. Appearance and pH Evaluation

KT hydrogel when freshly prepared showed a yellowish-translucent color, stickiness and limited flowability. The gel showed a neutral pH of 7.3. None of these aspects changed after two month-storage at room temperature.

#### 2.1.2. Optical Stability

The stability of the hydrogel was studied by observing if any change occurred in the backscattered light from the hydrogel using TurbiScanLab^®^ equipment. The analysis of changes in backscattered light, from the bottom to the top of the sample permits the detecting of sedimentation/creaming processes of colloidal dispersions, as well as coalescence/flocculation processes, with a much higher sensitivity as compared to the naked eye [17,18].

**Figure 2:**
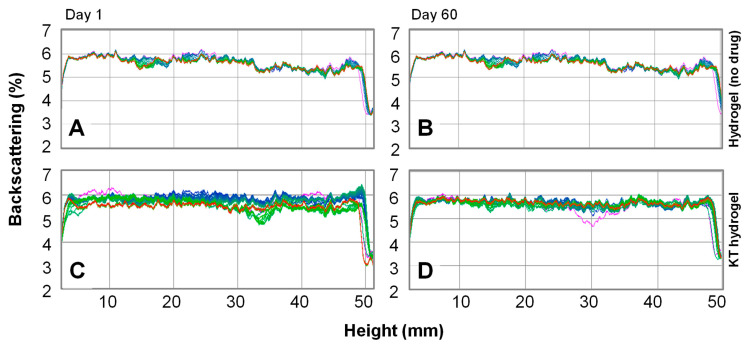
Hourly backscattering profiles over 24 h for (**A**) hydrogel without drug at day 1; (**B**) hydrogel without drug at day 60; (**C**) ketorolac hydrogel (KT hydrogel) at day 1; and (**D**) KT hydrogel at day 60. The color of the lines shows the hourly evolution within the day of analysis, being magenta the first and red the last analysis after the 24-h study.

The hydrogels (with ketorolac tromethamine and without the drug) freshly prepared were examined every hour for 24 h to observe potential changes in the short term. After two-months of storage at room temperature (23 ± 2 °C), the analysis was repeated to assess the stability in the long term. Figure 2 shows the backscattered light from a hydrogel without drug (A–B) and with ketorolac tromethamine (C–D), both at day 1 (A,C) and at day 60 (B, D), where the color of the lines shows the hourly evolution within the day of analysis, being magenta the first and red the last analysis after the 24-h study.

As it can be observed in the figure, both the hydrogel alone and with ketorolac tromethamine are mainly translucent, as the mean backscattered light is only around 6% in all cases, indicating that the inclusion of ketorolac tromethamine in the formulation does not affect the structure of the hydrogel and the drug remains in perfect solution. Moreover, neither the hydrogel alone nor the hydrogel with ketorolac tromethamine undergo sedimentation or creaming processes, as no significant increase/decrease in the backscattered light in the bottom/top of the sample is observed. For colloidal samples having a particle size bigger than 0.6 μm in diameter, flocculation is detected by the decrease of the mean backscattering values throughout the sample [17]. In this case, the 24-h evolution shows very little change (<1%) in the mean backscattered light of the freshly prepared KT hydrogel (day 1), suggesting a reversible restructuration of the gel fibers during the first 24 h, but with no evident flocculation process occurring, as the mean backscattered light on day 60 remained stable. These results therefore show that the KT hydrogel can be considered stable.

#### 2.1.3. Morphological Studies

**Figure 3:**
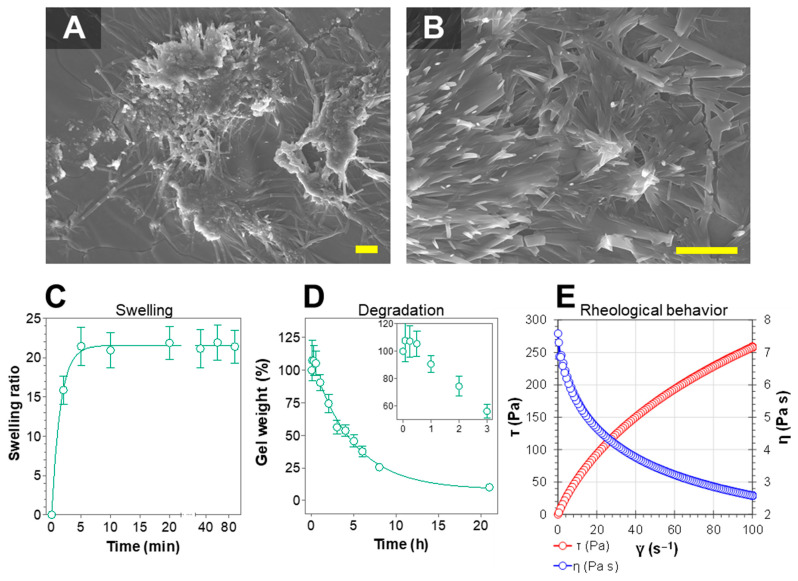
Characterization of KT hydrogel. (**A**,**B**) SEM images of KT hydrogel at different magnifications. The gel is formed by sodium alginate polymers that self-assemble to form fibers with diameters of around 100 nm. Scalebar represents 5 µm. (**C**) Swelling ratio of KT hydrogels upon immersion in PBS. The kinetics followed a first-order (one-phase exponential association) model (**D**) Degradation of KT hydrogels in PBS. Inset shows a 7% weight increase during the first hour, in accordance to swelling results. After one hour, degradation follows a one-phase exponential decay kinetics. Values represent Means ± SD (*n* = 3). (**E**) Rheogram of KT hydrogel at 32 °C. The flow curve represents shear stress (τ in Pa) (left axis) or viscosity (η in Pa·s) (right axis) as a function of the shear rate (γ in s^−1^).

Scanning Electron Microscopy (SEM) was used to evaluate the morphology of the KT hydrogel. Figure 3 shows that the gel is composed of sodium alginate polymer that self-assembles in the presence of water forming fibers that can be as thin as 80 nm in diameter. The sodium alginate-fibers can grow resulting in two different arrangements and therefore morphologies (Figure 3A,B). Fibers can grow longitudinally to several micrometers in length and can gradually attach to each other to form thicker fibers. Fibers of different thicknesses are intertwined and create pores with diameters ranging between 400 nm and up to one micrometer.

The high porosity as well as the hydrophilic behavior of the fibers is ideal from a drug delivery point of view, as it could permit the incorporation of solvent, and explains the high and fast swelling observed. In addition, it could predict a fast diffusion of the drug during drug release experiments (see below).

#### 2.1.4. Swelling and Degradation Tests

Swelling experiments were performed in KT hydrogels to evaluate their ability to incorporate solvent beneath the matrix. Briefly, previously dehydrated KT hydrogels were immersed in PBS, and their weight was recorded at certain intervals. Figure 3C shows that previously dehydrated gels are highly hygroscopic, as they can incorporate up to 20 times their weight in water in less than 5 min. The weight increase followed a first order kinetics (one-phase exponential association), with a constant value k = 0.68 min^−1^.

Moreover, degradation experiments were performed by immersing the KT hydrogels in PBS and recording their weight at certain intervals. Figure 3D shows an initial 7% weight increase during the first hour upon immersion, corresponding to the incorporation of PBS into the matrix, and which is in accordance with swelling experiments. After the first hour, the gel weight decreased following a first-order kinetics (one-phase exponential decay), with a degradation constant value of 0.21 h^–1^, therefore achieving 10% of the original weight after 21 h. This weight loss corresponds to the gradual diffusion of the components of the hydrogel into the medium, indicating that the formulation could be successfully degraded when immersed in the biological medium.

#### 2.1.5. Rheological Behavior

The mechanical properties of the KT hydrogel were studied by performing rheology studies, where the shear rate was first increased and subsequently decreased, while shear stress (τ) and the viscosity (η) were measured. The flow curve of KT hydrogels (Figure 3E) (*n* = 2) indicated no thixotropic behavior in the system as the rheogram did not display any hysteresis loop. The mathematical model that provided the best overall match of the experimental data according to the highest correlation coefficient of regression (r) was *Cross*, which indicates a shear thinning (pseudoplastic) behavior. Additionally, the results indicated that the viscosity of the different samples at 100 s^–1^ was 2555.5 ± 28.5 mPa·s, showing good repeatability of the rheological results between samples and indicating an adequate formulation protocol.

### 2.2. In Vitro Release of Ketorolac from the Hydrogel

**Figure 4:**
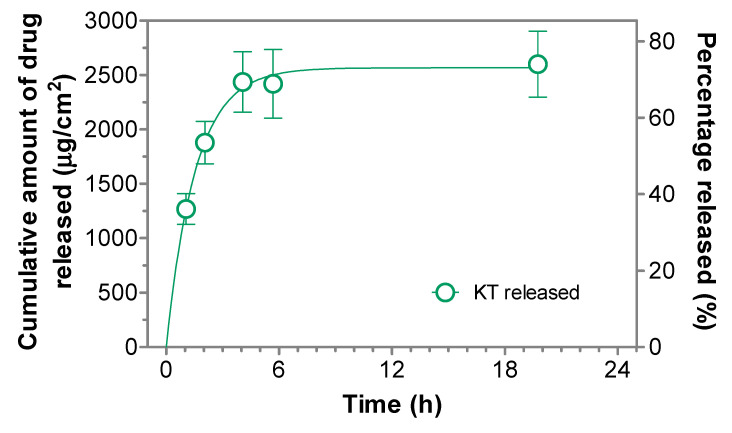
Cumulative amount of ketorolac released. Data followed a first-order (One-phase exponential association) kinetics. Values represent Means ± SD (*n* = 6).

In vitro drug release experiments were performed using Franz-type cells to evaluate the ability of the hydrogel to release the drug according to previously reported methodologies [19]. Figure 4 shows the cumulative amount of ketorolac released from the hydrogel. Different models were fitted (Zero-order, first-order and Higuchi) (see Appendix A, Table A1 and Table A2), and the best-fitting model was chosen according to the determination coefficient (r^2^). Ketorolac released from the hydrogel followed a first order (one-phase exponential association) kinetics, and the release parameters can be seen in Table 1.

Results show that the hydrogel can release up to 2568 µg/cm^2^ of ketorolac, corresponding to 73% of the total dose seeded, and this maximum amount is already achieved within 4 h, showing a release rate of 0.64 h^–1^. This fast drug release is in accordance with the wide and numerous pores created by the fibers arrangement, as observed in the SEM images (Figure 3A,B), which allow the easy diffusion of the drug.

### 2.3. Ex Vivo Permeation of Ketorolac through Human Skin and Vaginal Mucosa

**Figure 5:**
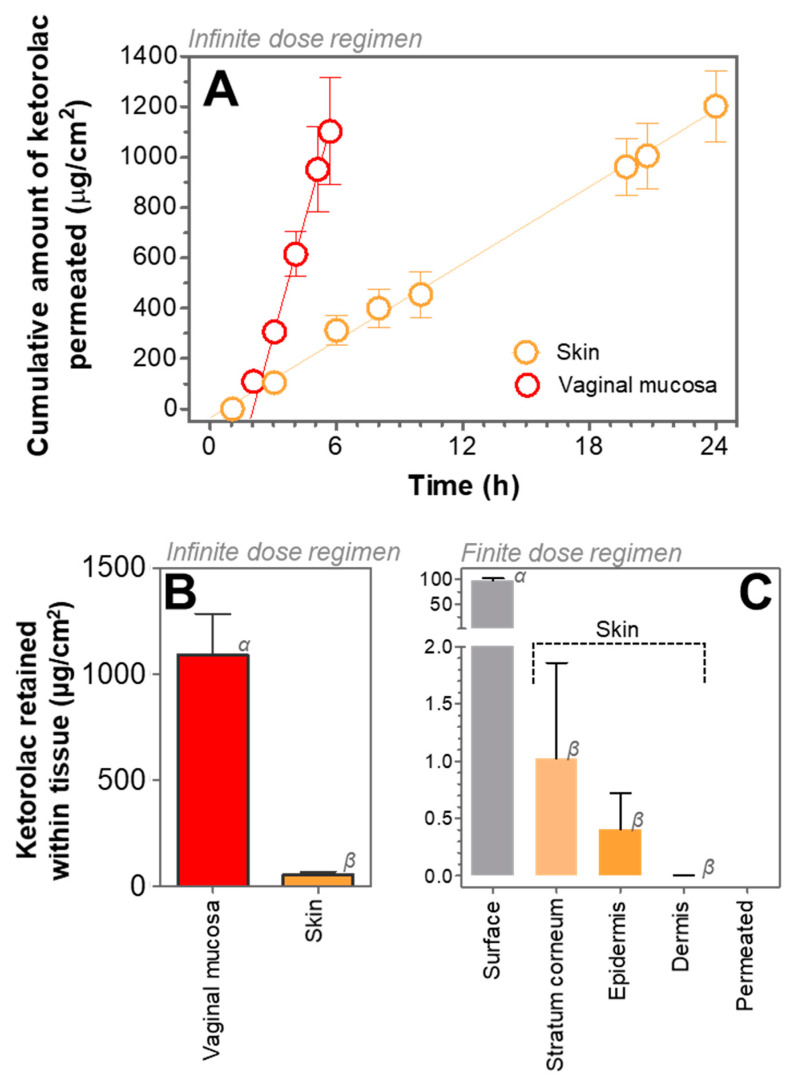
(**A**) Cumulative amount of ketorolac permeated (µg/cm^2^) through human skin and through vaginal mucosa upon application of KT hydrogel. (**B**) Total amount of ketorolac retained within the tissue (µg/g cm^2^) (skin or vaginal mucosa) 24-h after application of KT hydrogel under an infinite dose regimen. (**C**) Biodistribution of ketorolac 24 h after application of KT hydrogel using a finite dose regimen. Around 95% ketorolac remains outside the skin while the successfully penetrated ketorolac is retained within the stratum corneum and epidermis layers. All values represent Means ± SD (*n* = 6). Different Greek letters represent significant differences (*p* < 0.001).

Permeation experiments were performed ex vivo to assess the permeation of ketorolac across human skin as well as across vaginal mucosa according to previously reported methodologies [9,20,21], in both cases under an infinite-dose regimen [22]. The cumulative amount of ketorolac permeated through each tissue along time is shown in Figure 5A, and the permeation parameters calculated can be seen in Table 2.

Permeation of ketorolac across different tissues (skin or vaginal mucosa) upon application of KT hydrogel showed significant differences. In case of permeation across skin tissue, the permeation parameters indicate that ketorolac permeation reached the steady state from the very beginning, with an estimated T_lag_ of 0.67 h, and showing a transdermal flux of 50.92 µg/h·cm^2^, leading to a total amount of ketorolac permeated of 1202.46 µg/cm^2^ after 24 h. In contrast, permeation through vaginal mucosa indicated a slightly longer Tlag (2.04 h) while a significantly higher flux (306.00 µg/h·cm^2^) led to a similar amount of ketorolac permeated (1102.20 µg/cm^2^) in only 6 h.

These results can be explained by the fast release of ketorolac from KT hydrogel (Figure 4) as well as by the physicochemical properties of the drug (QSAR). For instance, it is considered that drugs with a molecular weight below 500 Da, an octanol: water partitioning coefficient (log P) below 5, less than 5 hydrogen bound donors, and less than 10 hydrogen bound acceptors, can successfully permeate through skin tissue [23,24]. These properties change when the drug is in its neutral form (ketorolac) or in the salt form (ketorolac tromethamine). For instance, the salt form has a higher molecular weight than the neutral form (376.4 vs. 255.27 g/mol), more hydrogen bond donors (5 vs. 1), more hydrogen bond acceptors (7 vs. 3), and although the log P for the salt form is not reported, it is expected to be much lower than that of the neutral form (1.9) [25,26]. It is true that the drug in contact with biological medium may change its form (and thus its properties), being in an equilibrium that depends on the concentration of the drug and the composition of the medium in which it is dissolved. Changes can include exchange of the cationic moiety (tromethamine) for other cations or protonation of the carboxylate group of ketorolac to a neutral carboxylic acid. Nonetheless, the use of the salt form in KT hydrogel may initially influence a higher proportion of ketorolac molecules in the salt form. For this reason, the faster permeation of ketorolac through the vaginal mucosa may be especially related to the highly hydrophilic behavior of the salt form (ketorolac tromethamine), as it does not find an important lipophilic barrier against the penetrating of the tissue and permits high fluxes across the mucosa. In contrast, permeation of ketorolac through the skin is compromised by the barrier effect produced by the epidermis (especially the highly lipophilic stratum corneum), leading to smaller fluxes. Examples of gels for topical application can be found in the literature showing that the physicochemical properties of the drug highly influence their ability to permeate through the skin. When applied using the same gel and at the same concentration, neutral, lipophilic drugs such as the corticoids betamethasone 17-valerate or triamcinolone acetonide tend to permeate faster than cationic drugs such as gemcitabine hydrochloride or anionic drugs such as methotrexate sodium [20].

Moreover, local anti-inflammatory activity with a rapid action onset may be achieved by a sufficient amount of drug retained in the tissue in short time. In this regard, short T_lag_ was observed in both tissues, and the total amount of ketorolac retained within the skin was 52.54 µg/cm^2^, whereas within vaginal mucosa was around 20 times higher (1090.42 µg/cm^2^). These results confirm the suitability of KT hydrogel for local application in either tissue but especially on vaginal mucosa, as the higher retention is most probably related to the higher contents of water in the tissue.

In order to assess a possible systemic effect after application of KT hydrogel under an infinite-dose regimen, either on skin or vaginal mucosa, the steady state plasma concentration (Css) after application of ketorolac gel was estimated, considering the human plasma clearance of ketorolac (1840 mL/h) [27] for a mean 80-kg individual. For instance, the expected Css achieved after application of these hydrogel considering a hypothetical area of application of 5 cm^2^ would be 0.22 µg/mL when applied on human skin, whereas 1.30 µg/mL when applied on vaginal mucosa. The therapeutic plasma concentration range reported is 0.3 to 5 µg/mL [28]; thus, the Css expected after a dermal application would be below the therapeutic plasma concentration range. On the contrary, the Css calculated after a 5 cm^2^ vaginal application would be within this reported range. However, no side effects would be expected since the Css is close to the lower limit. Additionally, topical products are not used in exaggerated use conditions (infinite dose approach) but in finite dose conditions. All this suggests that the KT hydrogel could be safely used for the local healing of inflammation. The equations for the calculation of Css and of the absorption parameters can be seen in the Appendix A (permeation parameters and calculation of the Css upon application of KT hydrogel).

### 2.4. Distribution of Ketorolac within the Skin Layers

A permeation experiment under finite dose regimen [22] was performed to study the skin biodistribution of ketorolac. For instance, around 10 µL of gel was applied on the skin, the dose of ketorolac trometamine being only 102 µg/cm^2^. After 24-h permeation, the receptor fluid was collected. Ketorolac remaining on the surface of the skin was carefully recovered, adhesive tape was used to perform stripping procedures to detach the *stratum corneum*, and the epidermis was then separated from the dermis by means of heat. Ketorolac contained in the adhesive tapes, the epidermis, and the dermis was extracted using a water:methanol mixture from the different layers and finally was analyzed (Figure 5C). Detailed absolute and relative amounts can be seen Table 3. After application of a small amount of KT hydrogel, most of the ketorolac could be recovered (92–101%). Approximately 95% of the dose applied was not absorbed, rather it was found on the surface, which can be related to the barrier effect produced by the highly lipophilic *stratum corneum* [29].

Regarding the drug penetrated into the skin, around 1% was found in the stratum corneum, 0.4% was found in the epidermis, and only 0.002% in the dermis, whereas no ketorolac was detected in the receptor fluid. This indicates no drug is absorbed into the systemic circulation when applying a small dose. This biodistribution suggests that upon application of small doses, finding the drug in the outer layers of the skin is not related to the affinity of the drug for these (lipophilic) layers, but is due to the low dose applied. In the beginning, the high drug concentration gradient at the surface of the skin promotes the diffusion inside the skin, but the low dose leads to a rapid decrease in the concentration gradient, impeding the further diffusion along the inner epidermis layers.

Therefore, the application of a small dose such as 10 µL on the skin would probably lead to anti-inflammatory activity in the outer layers of the skin only; however, when the KT hydrogel is applied in higher quantities, such as those used under the infinite dose regimen (~200 µL), it should be effective for the topical relieve of inflammatory processes, either applied through the skin or through vaginal mucosa.

### 2.5. In Vivo Anti-Inflammatory Efficacy Evaluation

To assess if the ketorolac retained within the skin after application (Section 2.3 and Section 2.4 ex vivo permeation experiments under finite and infinite-dose approaches) is sufficient to develop a local anti-inflammatory effect, an in vivo study was carried out in mice. Groups of mice (*n* = 6) were processed in parallel. In all groups, acute skin edema was induced upon application of TPA in both ears of the mice. At the same time, in one of the groups, KT hydrogel was applied to the right ear of the mice as a treatment, whereas 2% solution of ketorolac tromethamine in water was applied to the right ear in a second group, and finally, hydrogel without drug was applied to the right ear in a third group.

**Figure 6:**
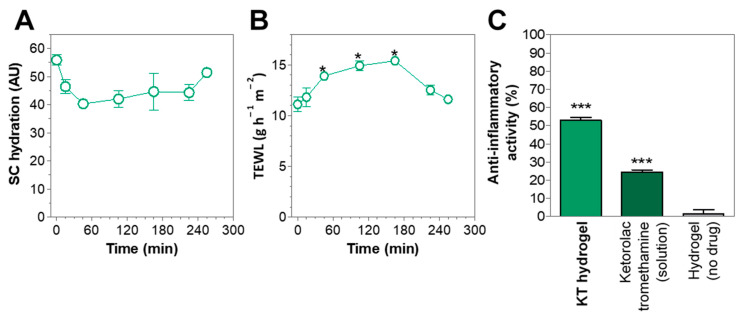
(**A**) Hydration of the *stratum corneum* upon application of KT hydrogel in humans. (**B**) Trans-epidermal water loss (TEWL) expressed in g of water per hour per m^2^ of skin, upon application of KT hydrogel in humans. (**C**) Anti-inflammatory activity expressed as inhibition (%) of TPA-induced inflammation upon application of a treatment (KT hydrogel, ketorolac tromethamine in solution, and hydrogel without drug). Values represent Means ± SD (*n* = 6). * Significant differences (*p* < 0.05); *** Significant differences (*p* < 0.001) as compared to control (hydrogel without drug).

The inhibition of inflammation achieved by the KT hydrogel as well as by the ketorolac tromethamine solution or the hydrogel without drug was calculated and is shown in Figure 6C.

The application of KT hydrogel led to a 52.9% reduction of inflammation as compared to the positive control, while application of ketorolac tromethamine in solution led to a 24.5% reduction of inflammation. However, KT hydrogel showed itself to be 2.16 times more effective than ketorolac tromethamine in solution, suggesting that the clinical application of KT hydrogel could be suitable for producing local anti-inflammatory activity.

### 2.6. In Vivo Tolerance Study

The preservation of the integrity of the skin upon application of KT hydrogel was studied using a corneometer. Parameters such as the hydration of the *stratum corneum* or the trans-epidermal water loss (TEWL) were analyzed and are shown in Figure 6A,B. Results show that upon application of the hydrogel, the basal hydration levels of the *stratum corneum* (55.9 ± 1.9 AU) decreased by 28%, reaching values of 40.3 ± 1.3 AU, 45 min after application, but gradually recovered to the initial values after 4 h. According to the normal values of skin hydration [30], values comprehended between 30 and 45 AU could be related to moderately dry conditions, while values above 45 AU could be related to a sufficiently hydrated skin. Thus, the application of KT hydrogel may result in only moderate dehydration, a condition that is reversible in the short term. The moderate dehydration of the stratum corneum could be explained by the highly hydrophilic behavior of the sodium alginate fibers forming the gel, that in contact with the stratum corneum may absorb the water from the surroundings, a behavior that was also observed in swelling experiments.

Likewise, TEWL is an indirect measure of skin permeability and the barrier function of the *stratum corneum* by observing the speed at which water is being evaporated from the surface [31]. It is generally accepted that TEWL values from patients with intact skin can range between 6 and 13 g h^–1^ m^–2^. In this study, untreated patients showed TEWL values of 11.1 + 0.7 g h^–1^ m^–2^, while upon application of KT hydrogel, TEWL values increased to 13.9 + 0.1 after 45 min and reached a maximum of 15.4 + 0.4 after 2.7 h but later decreased to basal values after 3.7 h. These results show that the application of KT hydrogel may slightly disrupt the barrier function of the stratum corneum but only to an extent which does not represent any threat to the physiological function of the skin, since TEWL values revert to the basal one in a few hours. The diffusion across a lipophilic structure, such as the stratum corneum, may be challenging for a highly water-soluble drug such as ketorolac tromethamine, and the hydrogel might be exerting a boost action on the Ketorolac’s penetration by modifying the vehicle-skin partition coefficient in a reversible manner by promoting the water content in the stratum corneum, and the water embedded in the hydrogel might drive the Ketorolac diffusion through the stratum corneum.

Studies have shown that skin variables such as TEWL, pH, and skin temperature in humans are dependent on circadian rhythms, interestingly finding that the skin permeability is higher during the evening and late night than in the morning [32]. Moreover, it has been shown in hairless rats that changes in the environmental temperature and light have an effect on skin function [33]. This suggests that the reversible TEWL values of the skin after application of KT hydrogel might be influenced by circadian rhythms and/or environmental factors such as temperature, and this suggests that its application during the evening might lead to higher analgesic and anti-inflammatory efficacy.

In addition, the application of KT hydrogel on patients’ skin did not lead to any visual irritation and was well tolerated.

## 3. Conclusions

An alginate-based hydrogel was extensively characterized in terms of morphology, pH, physical stability, and mechanical properties, showing adequate characteristics for a topical formulation.

Drug release studies show that up to 73% of the total amount of ketorolac in the gel can be released in less than 6 h from the hydrogel following the one-phase exponential model.

Permeation experiments under an infinite-dose approach were performed using human skin and vaginal mucosa, demonstrating that ketorolac contained in the hydrogel can permeate through both tissues successfully, showing almost 7 times faster permeation through vaginal mucosa (306.0 µg/h·cm^2^) than through human skin (50.92 µg/h·cm^2^). Moreover, ketorolac is retained within the tissue 24 h/6 h after application, showing an up to 20-times higher amount of drug retained within vaginal mucosa (1090.42 µg/cm^2^) than in human skin (52.54 µg/cm^2^).

The topical application of this hydrogel, in general, should not cause any systemic side effects, and therefore, it can be considered safe.

The hydrogel was well tolerated in vivo when applied in humans, showing no important alterations in the skin barrier function such as hydration and TEWL. Additionally, the hydrogel did not cause any visible irritation. These results show that KT hydrogel is a stable and suitable formulation for the treatment of inflammatory processes, such as those occurring upon chemical or surgical removal of anogenital warts.

## 4. Materials and Methods

### 4.1. Materials

#### 4.1.1. Reagents

Sodium alginate was purchased from Fagron Iberica (Terrassa, Spain). Ketorolac tromethamine and Nipagin were obtained from Sigma-Aldrich (Barcelona, Spain). Nipasol was acquired from Acofarma (Barcelona, Spain), and Na_2_HPO_4_ and KH_2_PO_4_ were supplied by Panreac (Barcelona, Spain). PBS pH 7.6, gentamicin sulphate, and bovine serum albumin were provided by Sigma (St. Louis, MO, USA); methanol was obtained from Merck, Darmstadt, Germany. The adhesive tapes were obtained from D-squame, (Cuderm Co., Dallas, TX, USA).

The purified water was obtained from a Milli-Q1 Gradinet A10 system apparatus (Millipore Iberica S.A.U., Madrid, Spain). All the other chemicals and reagents used in the study were of analytical grade.

#### 4.1.2. Tissues and Experimental Animals for Ex Vivo and In Vivo Assays

Human skin was obtained from abdominoplasties on healthy women (Barcelona SCIAS Hospital, Barcelona, Spain). The Bioethics Committee of the Barcelona-SCIAS Hospital approved the Study protocol (Nº001; approved on 20/01/2016), and volunteers provided written informed consent forms.

Following the surgery, the skin was stored at −20 °C until the experiments; then, the skin was dermatomed (GA630, Aesculap, Tuttlingen, Germany) at 500 μm-thick. The human skin barrier integrity of all skin discs was determined by transepidermal water loss (TEWL) using a TEWL-meter (Dermalab) prior to the permeation studies. Those skin discs that failed in the skin barrier integrity stage (TEWL values above 13 g/m^2^ h) were discarded and replaced.

Vaginal mucosa (Landrace Large White race) was provided by the Bellvitge animal facility services. The Ethics Committee of Animal Experimentation of the University of Barcelona approved the Study Protocol (approved on 10/01/2019). Full-thickness mucosa was used in the permeation studies.

Male Swiss CD-1 mice weighing 20–25 g (Círculo ADN S.A. de C.V.; Coyoacan D.F., Mexico) were used for the in vivo anti-inflammatory efficacy evaluation after a 7-day quarantine period. The animals were housed in plastic cages with soft bedding, controlled diet, and tap water ad libitum. Environmental conditions were controlled at 24 ± 1 °C, and the relative humidity was kept at 50–60%. Light conditions were also controlled, being 12 h light and 12 h dark every 24 h.

The study was performed according to the Mexican Official Normative for Animal Care and Handling (NOM-062-ZOO-1999), and the Academic Ethics Committee of the Vivarium at the Universidad Autónoma del Estado de Morelos (Mexico) approved the Study Protocol (BIO-UAEM:012:2013; approved on 21/12/2015).

### 4.2. Preparation of Sodium Alginate Hydrogels

Drug-loaded hydrogels were prepared at laboratory scale at a concentration of 2% *w*/*v* by dissolving ketorolac tromethamine in aqua conservans. Sodium Alginate (4%) was gradually added to the ketorolac tromethamine solution with continuous stirring, until a thin dispersion without residual powder was formed. The dispersions were allowed to equilibrate in a water bath at 37 °C for 24 h so that they would undergo the swelling process that led to the formation of a cross-linked polymeric network, obtaining KT hydrogel. The gel was stored at room temperature.

### 4.3. Physical Characterization of KT Hydrogel

#### 4.3.1. Appearance

The hydrogel formulation was visually observed directly for color, odor, and viscosity, at preparation and again 2 months afterwards.

#### 4.3.2. pH Measurements

The pH of the hydrogel was measured at room temperature using the pH meter micro-pH 200 (Crison Instruments S.A., Barcelona, Spain). Measurements were conducted with the gel freshly prepared and at 2 months after preparation.

#### 4.3.3. Optical Stability

The physical stability of the KT hydrogel was monitored by Dynamic Backscattering using a TurbiScanLab^®^ (Turbiscan T-Lab Expert (Formulaction Co., L’Union, France). The optical sensor comprises a pulsed near-infrared light source and two synchronous optical sensors: one for backscattering and one for transmission.

A volume of 20 mL of KT hydrogel was filled into flat-bottom cylindrical glass measuring cells, and backscattered light at λ = 880 nm was measured from the top to the bottom of the vial, hourly for 24 h at 25 °C to evaluate the short-term stability. Measurements were repeated two months later to assess the long-term stability. Hydrogel without drug was analyzed similarly as a control [34].

#### 4.3.4. Morphological Studies

The micro-structure of the hydrogel was examined by Scanning Electron Microscopy (SEM) in a JSM-7100F (JEOL Inc., Peabody, MA, USA) by coating the sample with a thin layer of carbon in an Emitech K950 coater (Quorum Technologies Ltd., Kent, UK).

#### 4.3.5. Swelling and Degradation Tests

The swelling test evaluates the hydrogel capacity of absorbing water within its structure. The PBS uptake was carried out in triplicate by determining the swelling ratio (SR) by a gravimetric method [9] according to Equation (1):

(1) $$SR=\;\frac{W_{s}\;-\;W_{d}}{W_{d}}$$

where *W_d_* is the weight of dried hydrogel, and *W_s_* is the weight of the swollen hydrogel at different times.

Briefly, the dried hydrogel was immersed in PBS (pH = 7.4) at 37 °C for 90 min. At different times (2, 5, 10, 20, 30, 60, and 90 min), the gel was removed from the incubation, and the excess PBS on the gel surface was soaked up and then weighed (*W_s_*).

The degradation test aims to monitor the weight loss as a function of time. The weight loss (WL) was calculated by incubating known amounts of fresh hydrogel in PBS (pH = 7.4) at 37 °C for 21 h. Three replicates of hydrogel were removed, blotted, and weighed at the following time points: 0.08, 0.25, 0.5, 1, 2, 3, 4, 5, 6, 8, and 21 h. The weight loss was expressed as the percentage of weight loss with respect to the freshly prepared hydrogel. It was calculated based on Equation (2):

(2) $$WL\left(\%\right)=\frac{W_{i}\;-\;W_{d}}{W_{i}}\;100\%$$

where *W_i_* is the initial weigh of hydrogel and *W_d_* the weight of hydrogel at different times.

#### 4.3.6. Rheological Behavior

The rheological properties of the sodium hydrogel containing 2% ketorolac tromethamine were determined by a rotational Haake RheoStress 1 rheometer (Thermo Fisher Scientific, Karlsruhe, Germany) equipped with cone-plate geometry (Haake C60/2° Ti, 60 mm diameter, 0.105 mm gap between cone-plate). Measurements were performed in duplicate at 32 °C (Thermo Haake Phoenix II + Haake C25P), the program consisted of a 3 steps shear profile: (1) a ramp-up period from 0 to 100 s^−1^ during 3 min, (2) followed by a constant shear rate period at 100 s^−1^ for 1 min, and (3) the ramp-down period from 100 to 0 s^−1^ for 3 min. Steady-state viscosity, determined at t_0,_ at 32 °C, was also calculated from the constant shear stretch at 100 s^−1^.

The flow data obtained were fitted to different mathematical models (Table 4) to describe the flow curve and characterize the flow properties:

### 4.4. In Vitro Release of Ketorolac from the Hydrogel

The in vitro release assay was conducted using amber glass Franz diffusion cells (Franz Diffusion cells (FDC 400, Crown Glass, Somerville, NY, USA) with an active diffusion area of 1.77 cm^2^ (*n* = 6). The receptor fluid consisted of 0.06M PBS (pH 7.4), which was continuously stirred with magnetic beads at 500 rpm to keep the contents of the receptor compartment homogeneous throughout the tests. The system was thermostatted at 37 ± 0.5 °C by a circulating water jacket. Sink conditions were held throughout the experiments, and 310 mg ± 10 mg of KT hydrogel was accurately applied to the membranes (mixed ester cellulose 0.45 µm pore size) in the donor compartment. Air bubbles entrapped below the membranes were removed, and the system was allowed to equilibrate for at least 30 min before applying the hydrogel. Parafilm was used to avoid evaporation by sealing the donor compartment and the sampling ports. Samples (300 µL) were collected at specific time intervals for about 20 h (1, 2, 4, 5.5, and 19.75 h), and the same volume was immediately replaced with PBS after the removal of each sample. The ketorolac was determined by High Performance Liquid Chromatography (HPLC) following the norms of a validated method, and the experimental data were fitted to different mathematical models (Zero-order, First-order, and Higuchi) (See Appendix A, Mathematical models fitted to drug release experiments). The best fitting model was chosen according to the correlation coefficient (r^2^) value.

### 4.5. Ex Vivo Permeation of Ketorolac through Human Skin and Vaginal Mucosa

The penetration and permeation of Ketorolac into and through the skin was evaluated by infinite dose and by finite dose approaches. The former provided information about the kinetics and permeation parameters (Section 4.5.1, Section 4.5.2 and Section 4.5.3), and the finite dose setup provided information about the distribution of Ketorolac within the skin, in its different layers with a realistic application approach (Section 4.6).

#### 4.5.1. Infinite Dose Approach Ex Vivo Permeation Assay in Human Skin

To study the rate and extend of Ketorolac that diffused through the skin, an ex vivo permeation assay was performed. The experiment was conducted as designed for the release assay except that 500-µm dermatomed frozen skin was used instead of synthetic membrane (*n* = 6). The skin was thawed at room temperature, and skin discs were punched and mounted on the Franz cell (0.64 cm^2^ of diffusion area) with the Stratum Corneum facing up to the donor compartment and the dermis in contact with the receptor fluid [35]. Hydrogel (200 mg) was applied to the skin, and samples (300 µL) were collected at the time intervals: 0, 3, 6, 8, 10, 19.75, 20.75, and 24 h and stored at −20 °C until sample analysis by HPLC was carried out.

#### 4.5.2. Infinite Dose Approach Ex Vivo Permeation Assay in Porcine Vaginal Mucosa

The ex vivo permeation test with full-thickness porcine vaginal mucosa aimed at investigating the capacity of ketorolac to diffuse through the vaginal tissue. It was conducted in the same manner as in Section 4.5.1, adjusting the sampling times to: 1, 2, 3, 4, 5, and 5.7 h. Samples were stored and analyzed by HPLC.

Results from the permeations assays fitted to a zero-order kinetic model, by means of a linear least squares regression, and the permeation parameters were calculated according to the equations described in Appendix A (Permeation parameters and calculation of the Css upon application of KT hydrogel).

#### 4.5.3. Amount of Ketorolac Retained in the Skin and in the Vaginal Mucosa

At the end of the permeation assays, the residual hydrogel on the skin or mucosa was removed by a swab, and the tissues were taken out of the Franz cells, cleaned with a gauze soaked in 0.05% solution of sodium lauryl sulfate and rinsed 3 times with distilled water. The permeation area of the skin/vaginal mucosa was then cut and weighed and perforated by a thin needle and incubated with 1 mL of methanol: water (1:1) to extract the Ketorolac remaining in the tissues. The mixtures were sonicated for 20 min., and the supernatants were pipetted and analyzed by the HPLC method, yielding the amount of ketorolac retained in the tissues.

### 4.6. Distibution of Ketorolac within the Skin Layers

The distribution of Ketorolac within the skin layers was assessed by ex vivo permeation assays in finite dose approach using human skin dermatomed at 500 µm. Skin discs were placed on thermostated (32 °C) Franz static diffusion cells (3 mL, 1.86 cm^2^ of exposed area, Lara-Spiral, Courtenon, France). The sink conditions were guaranteed throughout the assay by adding bovine serum albumin 1% (*w*/*v*) in the receptor fluid, which was PBS at pH 7.6, gentamicin sulphate 0.04% (*w*/*v*) was also added to prevent skin degradation [9]. The receptor fluid was under magnetic stirring throughout the assay. A volume of 10 μL of hydrogel was applied to the skin. Control cells were also used with placebo hydrogel to evaluate any potential interferences in the sample analysis of the receptor fluid samples or skin samples. After 24 h of exposition, the skin discs were demounted from the Franz cells and the residual hydrogel (either with or without Ketorolac) remaining on the skin surface was recovered. The receptor fluid was recovered, and the *stratum corneum* was isolated by the tape-stripping technique. Twelve strips were carried out to remove most of the *Stratum corneum*. The epidermis was separated from the dermis by heat treatment [9]. The skin samples were incubated in water:methanol (1:1, *v*/*v*) and sonicated for 20 min to extract the Ketorolac. The extraction solvent volume was 10 mL for the skin surface, 2 mL for the *Stratum corneum* and Epidermis, and 1 mL for the Dermis. The supernatants were pipetted and analyzed by the HPLC method. The amounts of ketorolac in each compartment are expressed as μg/cm^2^ and % of the applied dose.

### 4.7. Analysis of Ketorolac in Solution

The samples generated in the in vitro release and in vitro permeation studies were analyzed by a validated HPLC method. The chromatographic conditions were the following: the mobile phase consisted of acetonitrile (+0.065% triethylamine) and purified water (+0.165% acetic glacial acid), in an isocratic elution (1:1) at flux 1 mL/min. The column used was Symmetry C18 75 mm × 4.6 mm and 3.5 µm. The volume injected was 10 µL, and Ketorolac was determined at the wavelength of 314 nm.

### 4.8. In Vivo Anti-Inflammatory Efficacy Evaluation

The anti-inflammatory efficacy of the hydrogel was evaluated in accordance with the methodology described by Mallandrich et al. [9], which consisted of inducing an ear oedema inflammation on mice by 12-O-detradecanoylphorbol-13-acetate TPA. Swelling is caused by fluid accumulation in the interstitial tissue after vasodilation; this greater fluid retention, in turn, increases the weight of the ear, and it is correlated with the inflammatory/anti-inflammatory response.

Briefly, a TPA solution was prepared in ethanol at 0.05%. The animals were divided in 3 groups (*n* = 6), which were processed in parallel: group 1, KT hydrogel; group 2, 2% solution ketorolac tromethamine in water; and finally, group 3, hydrogel without drug. In all groups, acute skin edema was induced upon application of 5 μL TPA in the mice’s left ears, which served as 100% inflammation control. An equivalent volume of TPA was applied to the right ear of the animals and subsequently, 100 mg of formulation (KT hydrogel, ketorolac solution or placebo hydrogel, depending on the group) was also applied to the mice’s right ear as treatment.

At 4 h post-application, the animals were slaughtered, and 7-mm circular sections were cut from both ears and weighed to determine the anti-inflammatory activity. The inhibition of inflammation achieved by the KT hydrogel as well as by the ketorolac tromethamine solution or the hydrogel without drug was calculated according to the Equation (3):

(3) $$Inhibition\;\left(\%\right)=\left(\frac{Weight\;control\;ear\;-\;Weight\;treated\;ear}{Weight\;control\;ear}\right)\times 100$$

### 4.9. In Vivo Tolerance Study

Variations in the skins biomechanical properties were evaluated after the hydrogel application compared to the basal conditions. The parameters assessed were the Transepidermal water loss (TEWL–Dermalab), and the *Stratum corneum* hydration (SCH–Corneometer Courrage CM825). To this end, healthy subjects participated as volunteers in this study with prior approval of the Study Protocol by the Ethics Committee University of Barcelona (IRB00003099; approved on 20/03/2018). Six women—aged between 21 and 64—who gave their informed consent were asked to abstain from the application of cosmetics 6 h prior to the test. They had a 20 min adaption period to acclimate themselves to the room conditions before each measurement. Prior to the hydrogel application, the site of application was marked by a template, and the basal values of TEWL and SCH were recorded for the test areas. Then, the hydrogel was applied homogenously as a thin layer to each volunteer’s left forearm. The following readings were performed at different time points for about 4 h (15, 45, 105, 165, 225, and 255 min).

### 4.10. Statistical Analysis

The cumulative amounts of the drug released and permeated over time were determined using linear regression analysis. The influence of the KT hydrogel on the TEWL and hydration of the skin was analyzed by one-way analysis of variance (ANOVA) at the time points. The significance level was set to 0.05.

GraphPad Prism^®^, v. 5.00 software (San Diego, CA, USA) was used for all statistical calculations.

## Figures and Tables

**Table 1:** Drug release parameters from KT hydrogel according to a first order kinetics. Ymax = total amount of drug released; K = release rate constant. Values represent Means ± 95% confidence interval (*n* = 6).

Best-Fit Values	
Ymax (µg/cm^2^)	2568 ± 14
K (h^–1^)	0.64 ± 0.12
Half-time (h)	1.08
R^2^	0.9908

**Table 2:** Transdermal/transmucosal permeation parameters of ketorolac 24 h/6 h after application of KT hydrogel under an infinite dose regimen. Results are expressed as Mean ± SD (*n* = 6). A_P_: amount of ketorolac permeated after 24 h/6 h. A_R_ amount retained after 24 h/6 h. Css: plasma concentration at steady state. Tlag: lag time. Jss: transdermal/transmucosal flux.

	Skin	Vaginal Mucosa
A_P_ (µg/cm^2^)	1202.46 ± 143.99	1102.20 ± 212.08
A_R_ (µg/cm^2^)	52.54 ± 14.71	1090.42 ± 196.27
Css (µg/mL)	0.22 ± 0.01	1.30 ± 0.05
T_lag_ (h)	0.67 ± 0.003	2.04 ± 0.50
Jss (µg/h·cm^2^)	50.92 ± 1.44	306.00 ± 6.95
K_p_ (cm/h)	2.55 × 10^–3^	1.53 × 10^–2^

**Table 3:** Distribution of ketorolac within the skin layers 24 h after application of KT hydrogel under a finite-dose regimen. b.l.q. stands for ‘below limit of quantification’

KT Hydrogel Application	µg/cm^2^	%
Ketorolac trometamine dosed	102.09	100.00
Surface	96.72 ± 4.07	94.75 ± 3.99
Stratum corneum	1.02 ± 0.84	1.00 ± 0.82
Percutaneous absorption	0.40 ± 0.32	
Epidermis	0.40 ± 0.32	0.39 ± 0.32
Dermis	0.002 ± 0.001	0.002 ± 0.001
Receptor fluid	b.l.q.	b.l.q.
Total recovery	98.14 ± 4.69	96.14 ± 4.60

**Table 4:** Mathematical models for the flow characterization by regression analysis.

$\mathrm{Flow}\;\mathrm{Curve}—\mathrm{Models}:\;\tau =f\left(\dot{\gamma }\right)$	
Newton	$\tau =\eta \cdot \dot{\gamma }$
Bingham	$\tau =\tau_{0}+(\eta_{0}\cdot \dot{\gamma )}$
Ostwald–de Waele	$\tau =K\cdot {\dot{\gamma }}^{n}$
Herschel–Bulkley	$\tau =\tau_{0}+K\cdot {\dot{\gamma }}^{n}$
Casson	$\tau =\sqrt[{n}]{\left(\tau_{0}^{n}+{\left(\eta_{0}\cdot \dot{\gamma }\right)}^{n}\right)}$
Cross	$\tau =\dot{\gamma }\cdot (\eta_{\infty }+(\eta_{0}-\eta_{\infty })/(1+{\left(\dot{\gamma }/{\dot{\gamma }}_{0}\right)}^{n})$

where, *τ* is the shear stress (Pa), *η* is the dynamic viscosity (mPa·s), $\dot{\gamma }$ is the shear rate (1/s), *τ*_0_ is the yield shear stress (Pa), *η*_0_ is the zero-shear rate viscosity, *η*_p_ is a constant plastic viscosity (mPa·s), *η_∞_* is the infinity shear rate viscosity, *n* is the flow index, and *K* is the consistency index. The goodness of fit was determined by correlation coefficient (r) by linear regression analysis of the flow plots.

## Data Availability

The data presented in this study are available on request from the corresponding author. The data are not publicly available due to they take part in a Doctoral Thesis, and they will be available once the Thesis will be published.

## Abbreviations

°C	Degree Celsius
µL	Microliter
µm	Micrometer
ANOVA	One-way analysis of variance
AP	Amount ok ketorolac permeated
AR	Amount ok ketorolac retained
C_0_	Initial concentration
CA	California
CA	Condyloma acuminata
Clp	Human plasma clearance
cm	Centimeter
cm^2^	Square centimeters
CO_2_	Carbon dioxide
Css	Plasma concentration at steady state
Da	Dalton
g	Gram
h	Hour
HPLC	High Performance Liquid Chromatography
HPV	Human Papilloma Virus
J	Flux
Jss	Flux at steady state
K	Release rate constant
Kg	Kilogram
KH_2_PO_4_	Potassium dihydrogen phosphate
Kp	Permeability coefficient
KT	Ketorolac tromethamine

## Appendix A

### Appendix A.1. Mathematical Models Fitted to Drug Release Experiments

The amount of ketorolac released from the gels was fitted to the following model equations [36]:

**Table A1:** Equations of the kinetic models fitted.

Zero-order	Rt=R∞+Ko×t
First-order	$Rt=R\infty \times \left(1-e^{k\times t}\right)$
Higuchi	$Rt=R\infty +Kh\times t^{1/2}$

where *Rt* is the amount of drug released at time *t*, *R∞* is the maximum amount of drug released, *K* is the release rate constant expressed in units of concentration/time, and *n* is the diffusion release exponent that can be used to characterize the different release mechanisms. It has been established that *n* ≤ 0.43 (Fickian diffusion mechanism), 0.43 < *n* < 0.85 (anomalous transport) and *n* ≥ 0.85 (Super case II transport, i.e., zero, order release) [36].

The best fitting model was chosen according to the correlation coefficient (r^2^) value (Table A1), for which drug release was determined to follow a First-order (one-phase exponential association) kinetics.

**Table A2:** Correlation factors of the fitted mathematical models.

	R^2^
Zero-order	0.4380
First-order	0.9908
Higuchi	0.6000

### Appendix A.2. Permeation Parameters and Calculation of the Css upon Application of KT Hydrogel

The steady state plasma concentration (Css) was calculated assuming an area of application of 5 cm^2^ according to the Equation (4):

(4) $$\mathrm{Css}=\frac{J\cdot \mathrm{TSA}}{\mathrm{Clp}\cdot A}$$

where Css is the steady state plasma concentration (Css), J (µg/h) is the flux, TSA (cm^2^) is the theoretical surface area of application, Clp (mL/min) is the human plasma clearance of ketorolac, and A (cm^2^) is the diffusion area of the Franz cells. An area of application of 5 cm^2^ was assumed.

The permeability coefficient was calculated in accordance with the Equation (5).

(5) $$\mathrm{Kp}=\frac{J}{C_{0}}$$

where Kp is the permeability coefficient of ketorolac through the membranes; J (µg/h) is the flux, and C_0_ (µg/mL) is the initial concentration of ketorolac in the gel.
